# Analyzing Primary Healthcare Governance in Indonesia: Perspectives of Community Health Workers

**DOI:** 10.7759/cureus.56099

**Published:** 2024-03-13

**Authors:** Mubasysyir Hasanbasri, Ahmad W Maula, Bayu S Wiratama, Aufia Espressivo, Tiara Marthias

**Affiliations:** 1 Department of Biostatistics, Epidemiology, and Population Health, Universitas Gadjah Mada, Yogyakarta, IDN; 2 Center for Health Policy and Management (CHPM), Universitas Gadjah Mada, Yogyakarta, IDN; 3 Research and Policy Division, Centre for Indonesia’s Strategic Development Initiatives (CISDI), Jakarta, IDN; 4 Nossal Institute for Global Health, Melbourne School of Population and Global Health, The University of Melbourne, Melbourne, AUS; 5 Department of Health Policy and Management, Universitas Gadjah Mada, Yogyakarta, IDN

**Keywords:** primary healthcare, health system determinants, community health, community health workers, governance roles, village heads, posyandu, community health posts

## Abstract

Background

Community-integrated health posts (Posyandu) are crucial for extending primary healthcare across diverse geographical and demographic landscapes in Indonesia. Community health workers (CHWs) currently function as the main service delivery actors for Posyandu. However, Posyandu’s performance remains below the standards set by the Ministry of Health. This study examines health system determinants that explain the poor performance of Posyandu and, in particular, examines the roles of village and township governance and community health center management in supporting the effectiveness of primary healthcare programs.

Methodology

We analyzed 638 Posyandus across 13 Indonesian provinces, utilizing data from the 2014 Indonesia Family Life Survey. We evaluated eight health system determinants based on the perceptions of CHWs concerning challenges encountered in Posyandus. These factors were ranked and analyzed to determine the variables that affected Posyandu’s poor performance. Both unadjusted and adjusted odds ratios were calculated.

Results

This study revealed that nearly half of the Posyandus in Indonesia are underperforming, particularly in rural areas. Common challenges include insufficient funds, inadequate equipment, and a lack of permanent buildings. Although only a small percentage mentioned minimal support from village and Puskesmas authorities, the weak governance roles of village heads are reflected in all the concerns mentioned by CHWs.

Conclusions

The absence of village heads from governance roles has contributed to Posyandu’s operational problems as perceived by CHWs. Community health centers (Puskesmas), responsible for providing technical support to Posyandu, should be part of CHW teams and networks. Further discussions are needed to choose a workable governance model to ensure practical, accessible, and sustainable primary healthcare services at the grassroots level.

## Introduction

The Astana Declaration of 2018 aimed to reinvigorate primary healthcare (PHC) efforts, emphasizing community-based services that align with essential public health and health equity functions [1,2]. Redirecting healthcare toward primary care is linked to achieving the Sustainable Development Goals, and examples from Thailand and India showcase successful transitions to community-level care [3]. However, building comprehensive PHC systems faces challenges. These include the lack of sustained political commitment and financing models [4,5]; insufficient health workforce recruitment, retention, and competence [6,7]; insufficiently addressing community needs [8,9]; and conflicts and disease outbreaks [9,10], as seen during the recent pandemic. A lack of political commitment may also lead to weak governance in PHC.

Indonesia, recently classified as an upper-middle-income country [11], with the fourth largest population and one of the fastest-growing economies globally, has made notable progress in improving health outcomes. However, challenges persist in remote regions and have been addressed through strategies such as mobilizing health workers and scaling national health insurance. Public community health centers (Pusat Kesehatan Masyarakat or Puskesmas) are primarily responsible for leading the PHC network in Indonesia. Puskesmas are crucial for providing comprehensive care, particularly in remote areas. Recent changes to the Indonesian health system include primary care transformation, empowering community health workers (CHWs) to strengthen public health services, primary and secondary preventive healthcare, and quality primary health services [12]. Core to the Puskesmas network is the community-based integrated health post (Pos Pelayanan Terpadu or Posyandu), which operates as a community-based health service. The significance of Posyandu in Indonesia cannot be overstated, as it serves as a crucial frontline service for a country where much of the population lives in remote areas far from the reach of conventional PHCs.

PHC systems and the governance of Posyandu in Indonesia

The PHC system in Indonesia is mainly led by public healthcare centers (Puskesmas). Posyandus is the public health outreach of Puskesmas, providing promotional and preventive health services down to the community level, consisting of five core services, namely, family planning, maternal and child health services, immunization, child nutritional programs, and diarrhea management. The Indonesian government has also set out to strengthen Posyandu to assist Indonesians of all ages in institutionalizing healthy lifestyles and facilitate access to primary care physicians as needed [13].

CHWs, primarily women residing in Posyandu catchment areas, assist Puskesmas staff in organizing Posyandu activities. Posyandu operates once a month to monitor the weight of toddlers to detect early growth delays, either because of malnutrition or treatable infectious diseases [14,15]. However, Posyandus’ capacity and ability to deliver health services vary across Indonesia. For instance, some Posyandus cannot meet the minimum yearly requirement of at least eight activities. Other Posyandus can provide more comprehensive and routine services [16,17].

The Ministry of Health classifies Posyandu into four strata to encourage community health centers and village governments to increase community participation in Posyandu [18]. The strata rely heavily on the number of active CHWs, permanent places of service, and medical supplies. For example, active CHWs, who are key community health volunteers, are crucial for the effective delivery of essential health services, community engagement, and ensuring the smooth operation of Posyandu. However, the lack of a permanent service place indicates the inability to provide consistent and reliable health services to the community.

The government of Indonesia has planned a rather complex organizational structure for Posyandu, partly to empower districts to take on more responsibilities in the public health area. While village heads organize decentralized community resources and involvement in implementing Posyandu, Puskesmas manages activities involving health professionals [15]. In Indonesia’s decentralized system, village heads are under the Ministry of Home Affairs, while Puskesmas is under the Ministry of Health. This complex arrangement of Posyandus requires careful examination and strategies to improve its governance, particularly for decision-making and resource management, which are the two main factors of governance [18,19], including for Posyandu.

Research on Posyandu is growing [19,20], but there is a need for a broader perspective on health systems, particularly from the governance aspect of Posyandu. This is crucial in a decentralized health system such as Indonesia, where the district government oversees many health issues while aligning with vertical programs that still dominate the PHC network.

Despite previous research evaluating the benefits of specific programs conducted by Posyandu, there remains a significant gap in our understanding of the PHC system that supports Posyandu activities. Most existing studies focus on the outcomes of individual Posyandu programs rather than on the overarching system that underpins these community health efforts [20,21]. This lack of comprehensive analysis creates a critical gap in the literature, particularly in understanding how the system evolves and adapts to support effective community health initiatives.

This study aims to bridge this gap by examining the Posyandu development status, defined by the Ministry of Health, from the perspective of CHWs and the data available as part of the Indonesian Family Life Surveys (IFLS) of Rand Corporation, based on their experiences in implementing programs at Posyandu, offering a unique perspective that has been largely overlooked. Following the health system and community health governance framework, this study contributes to Indonesia’s academic discourse on PHC systems. It offers practical insights for policymakers and practitioners aiming to enhance the effectiveness of community health programs and inform countries about integrating community-based health services into broader health systems.

## Materials and methods

Data

We used data from the latest wave of the IFLS, which was conducted in 2014 (Wave 5). The IFLS is an ongoing longitudinal survey that started in 1993 with four subsequent rounds of data collection (1997-1998, 2000, 2007-2008, and 2014-2015). The IFLS also includes surveys on health facilities, which encompass information on public primary health facilities, including Puskesmas and community sub-health centers (Puskesmas Pembantu); Posyandu; and private primary health facilities, which consist of private clinics, individual practices of primary care physicians, and nurses/midwives. Within each stratum, a maximum of five private facilities and three public facilities were selected, reflecting the typically greater number of private providers. Within IFLS Wave 5, 709 Posyandus were included across Indonesia. After considering missing values, the final sample was 638. The data comes from the “pos.dta” module of the community facility data available from the survey website.

Dependent Variable

“Poorly performing Posyandu” was coded as 1 if it was an early-stage or intermediate-stage Posyandus, while “advanced and self-sufficient Posyandus” was coded as 0. (See Supplementary File 1 in Appendices for the Posyandu development status used in the data.)

Independent Variables

The health system determinant variables included eight Posyandu problems perceived by CHW respondents in the survey. We created the following two groups: the village head’s responsibility comprises (a) lack of support from the village, (b) lack of interest/participation, (c) lack of active CHWs, (d) lack of funds, and (d) no permanent place (the absence of a permanent service location). Puskesmas responsibility covers (a) inadequate Puskesmas support, (b) a shortage of logistics and materials, and (c) a lack of equipment. These categories and their specific challenges are described in Supplementary File 1 in Appendices.

Table 1 shows the mapping of health system building blocks [22], aspects related to how Posyandu is organized and governed, and perceptions of CHWs of their challenges based on the IFLS questionnaire.

**Table 1:** Health system blocks, governance roles, and CHWs’ perceived problems of Posyandu. CHWs = community health workers; IFLS = Indonesia Family Life Survey

Health systems approach	Posyandu’s perceived problems	Governance roles		Descriptions/Activities
		Village	Puskesmas	
Outcome/Target population	Community interest or participation	Yes	Yes	Campaign to promote health literacy by coming to the Posyandu. Invite community activists to organize collective works in Posyandu. Assign Puskesmas workers to promote community-based health improvement activities
Service delivery	Active CHWs	Yes	Yes	Village staff should invite active community members to serve as CHWs. Motivate the community to take action to prevent diseases and promote healthy lifestyles. Seek non-financial incentives to make CHWs love to work for their community. Contract community midwives or nurses to work with CHWs to manage effective programs
Service delivery	Permanent service place	Yes	No	Posyandu services are usually at the village administrator’s house. Village heads should take action if no one allows their house to be used as a service place
Reporting and information systems	Not mentioned in the IFLS	Yes	Yes	Simple recording and reporting forms and systems accessible for monitoring Posyandu’s activities
Human resources	Not mentioned in the IFLS	Yes	Yes	Actively recruit (voluntary or paid basis) and ensure the engagement of CHWs on health programs. Support tailored health service training and other capacity-building activities for CHWs
Financing	Funding support	Yes	No	Allocate village funds for operational support of monthly services, such as snacks and drinks for monthly sessions, additional food for under-fives, copies of leaflets, weighing equipment, tables, and chairs for five basic services, chairs or mats, posters, and books for learning.
Logistics	Medical supply	No	Yes	There has been no involvement from the village because it is part of Puskesmas of the Ministry of Health
	Equipment	Yes	No	Equipment, including scales and blood pressure, is the village’s responsibility
Governance	Support from the village	Yes	No	Manage public resources and community participation. Propose an annual bottom-up plan (Musyawarah Perencanaan Pembangunan, or Musrenbang)
Governance	Support from Puskesmas	No	Yes	Assign nurses, midwives, health promotion, or nutritionists to supervise and work with CHWs in Posyandu activities

Statistical analysis

We utilized cross-tabulation and presented the results as frequencies and percentages in the univariate phase to describe the data distribution. Using simple logistic regression, bivariate analysis was used to measure the associations between covariates and outcome variables, and the results are presented as crude odds ratios. Multiple logistic regressions were used to calculate adjusted odds ratios. A p-value less than 0.05 in the Wald chi-square test of logistic regression showed a significant association between variables. To examine the consistency of the effects of our variables of interest across different settings, we stratified our analysis by rural-urban areas and regional divisions within Indonesia (Java versus other Java islands). For all statistical analyses, we used STATA version 15 from StataCorp (2017).

## Results

CHW perceived problems with poor-performing Posyandus

This study highlights two main PHC issues at the local level in Indonesia. First, there is a mismatch in what is more important - daily work problems or management issues. CHWs believe that difficulties with day-to-day operations are more critical compared to more significant management issues (see Table 2). The most significant problems CHWs perceived include not having enough funding for Posyandu activities (57.7%), not having enough equipment (37%), having no fixed place for Posyandu activities (27.9%), and having low involvement from the local community (23.5%). Less attention is given to management issues, such as insufficient support from local leaders (10.3%) and health centers (Puskesmas) (5.8%).

**Table 2:** Characteristics of Posyandu based on urban-rural and Java-outside Java contexts. CHWs = community health workers

Variables	Urban (n = 428) %	Rural (n = 210) %	Java (n = 417) %	Outside Java (n = 131) %	All (N = 638) %
Posyandu development status					
Early stage (Pratama)	12.6	19.5	14.9	14.9	14.9
Intermediate (Madya)	27.3	27.6	26.1	29.9	27.4
Advanced (Purnama)	28.7	25.7	27.3	28.5	27.7
Self-sufficient (Paripurna)	23.6	15.2	22.5	17.7	20.9
Not known	7.7	11.9	9.1	9.1	9.1
% of poor-performing Posyandu (Pratama and Madya)	39.9	47.1	41.0	44.8	42.3
Challenges perceived by Posyandu CHWs in the last two years					
Village/Township head governance					
Lack of fund	53.3	66.7	58.3	56.6	57.7
No permanent place	26.2	31.4	27.6	28.5	27.9
Lack of interest/Participation	24.1	22.4	22.1	26.2	23.5
Lack of active CHWs	10.3	20.0	13.4	13.6	13.5
Lack of support from the village	8.9	13.3	8.9	13.1	10.3
Puskesmas governance					
Lack of equipment	33.9	43.3	37.4	36.2	37
Lack of medical supply	20.1	18.6	20.9	17.2	19.6
Lack of support from Puskesmas	4.7	8.1	6.0	5.4	5.8

Second, while active CHWs are critical to PHC activities at the community level, around 13.5% of CHWs perceived that there were insufficient active voluntary workers with a higher percentage in the rural area (20.0%).

Rural contexts

The rural-urban context plays a significant role in Posyandu’s varying performance, as evidenced by the marked differences between these environments. Notably, a substantial proportion of Posyandus were underperforming, with 42.3% identified as such. This issue is more pronounced in rural areas, where 47.1% of Posyandus fall into the poor-performing category compared to 39.9% in urban settings. The inferior performance of Posyandus in rural areas is consistent, particularly in specific domains such as receiving support from village heads and Puskesmas, where rural areas lagged behind their urban counterparts in six out of eight evaluated areas. The shortages are consistently greater in rural areas than in urban areas, except for two aspects, i.e., lack of interest or participation and medical supplies.

Outside the Java context

Although the difference is small, Posyandus outside Java are in a worse state than those outside Java. Outside of Java, Posyandus have a greater percentage of problems related to the role of village heads and community interest and participation.

Determinants of poor-performing Posyandus

Table 3 shows the distribution of factors using bivariate analysis. Poor-performing Posyandus had higher odds of having a lack of active CHWs (odds ratio (OR) = 2.6, 95% confidence interval (CI) = 1.6-4.2) than good-performing Posyandus. The analysis also showed that poor-performing Posyandus had a two times higher probability of having a lack of support from Puskesmas (OR = 2.4, 95% CI = 1.2-4.7) than good-performing Posyandus. Other significant factors of poor-performing Posyandus in bivariate analysis were lack of funds, no permanent service place, lack of medical supply, lack of equipment, and location in rural areas.

**Table 3:** Adjusted odds ratios of poor-performing Posyandu determinants. *: significant at 10%; **: significant at 5%; ***: significant at 1%. CHWs = community health workers

Independent variables	Indonesia		All (N = 638)
	Urban (n = 428)	Rural (n = 210)	
Village/Township governance variables			
Lack of support from the village	1.3 (0.6-2.7)	1.0 (0.4-2.5)	1.1 (0.6-1.9)
Lack of interest or participation	1.2 (0.7-1.9)	0.9 (0.4-1.7)	1.1 (0.7-1.6)
Lack of active CHWs	2.0** (1.0-4.0)	2.8*** (1.3-5.9)	2.3*** (1.4-3.7)
Lack of fund	1.1 (0.7-1.7)	1.1 (0.6-2.2)	1.1 (0.8-1.5)
No permanent place	1.5* (1.0-2.4)	1.4 (0.8-2.6)	1.5** (1.0-2.1)
Puskesmas management variables			
Lack of support from Puskesmas	3.37** (1.21-9.41)	1.0 (0.3-3.3)	1.8 (0.9-3.8)
Lack of medical supply	1.90** (1.12-3.23)	0.7 (0.3-1.5)	1.3 (0.9-2.1)
Lack of equipment	1.17 (0.74-1.83)	0.9 (0.5-1.7)	1.1 (0.8-1.6)
Rural (vs. urban)	-	-	1.2 (0.8-1.6)

Table 4 shows the results from multivariate analysis. After controlling for other factors, the absence of active CHWs significantly affects the performance of Posyandus (adjusted odds ratio (aOR) = 2.3, 95% CI = 1.4-3.7), with a similar impact observed in both villages (aOR = 2.0, 95% CI = 1.0-4.0) and cities (aOR 2.8 = 95% CI = 1.3-5.9). Another key point is that Posyandus lacking permanent service buildings were more likely to have poor performance (aOR = 1.5, 95% CI = 1.0-2.1) than Posyandus with permanent service places. The effect of lacking a permanent service place is higher in urban than rural. In terms of governance, the lack of support from Puskesmas had a much greater impact in urban (aOR = 3.4, 95% CI = 1.2-9.4) than in rural (aOR = 1.0, 95% CI = 0.3-3.3) or all type of Posyandus (aOR = 1.8, 95% CI = 0.9-3.8).

**Table 4:** Non-adjusted odds ratio of poor-performing Posyandu determinants. *: significant at 10%; **: significant at 5%; ***: significant at 1%. CHWs = community health workers

Problems perceived by Posyandu CHWs in the last two years	Good-performing Posyandu (n, %)		Poor-performing Posyandu (n, %)		Total (n, %)		Odds ratio (95% CI) for poor-performing Posyandu	
Village/Township governance								
Lack of support from village or township	32	(8.7)	34	(12.6)	66	(10.3)	1.5	(0.9-2.5)
Lack of interest or participation	83	(22.6)	67	(24.8)	150	(23.5)	1.1	(0.8-1.6)
Lack of active CHWs	32	(8.7)	54	(20.0)	86	(13.5)	2.6***	(1.6-4.2)
Lack of funds	202	(54.9)	166	(61.5)	368	(57.7)	1.3*	(0.9-1.8)
No permanent service place	90	(24.5)	88	(32.6)	178	(27.9)	1.5**	(1.1-2.1)
Puskesmas management governance								
Lack of support from Puskesmas	14	(3.8)	23	(8.5)	37	(5.8)	2.4**	(1.2-4.7)
Lack of medical supply	61	(16.6)	64	(23.7)	125	(19.6)	1.6**	(1.1-2.3)
Lack of equipment	125	(34.0)	111	(41.1)	236	(37.0)	1.4*	(1.0-1.9)
In rural areas	111	(30.2)	99	(36.7)	210	(32.9)	1.3*	(1.0-1.9)
In outside Java	122	(33.2)	99	(36.7)	221	(34.6)	1.2	(0.8-1.6)

## Discussion

This is the first study to evaluate Posyandu’s performance as a community-level PHC network that uses the health systems lens, with an emphasis on governance and leadership factors. The findings of this study carry significant implications for strengthening the policy on integrated primary care in Indonesia, particularly concerning the performance of Posyandu and the roles of governance actors at the village and district levels. Posyandu plays a vital role in extending PHC services across diverse landscapes in Indonesia, yet this study reveals that nearly half of Posyandus are underperforming, especially in rural areas. Common challenges identified include insufficient funds, inadequate equipment, and a lack of permanent buildings.

The importance of CHWs in Posyandu service delivery

In the context of health systems frameworks, service delivery is the top-priority building block for ensuring continuity of care [22]. It is, therefore, paramount to address the efficiency and effectiveness of public health programs as part of service delivery. Our research has identified interrelated issues among three actors that may negatively affect service quality: (a) deficiencies in active CHWs; (b) a lack of support from Puskesmas; and (c) a lack of leadership among village heads. We consider the concept of governance to be a crucial element in PHC systems, represented by Puskesmas and official community administrators [23,24]. This indicates that systems thinking and strengthening frameworks help us capture the roles of Puskesmas and village heads.

The interrelationship can be explained as follows: CHWs are less motivated to participate without recognition from Puskesmas and village heads. Similarly, village heads may not be interested in governance because they might consider other activities more of a priority. Likewise, health workers who are overloaded daily may not have time to consider engaging in community activities. This shows that the issue is not merely individual or limited to implementation but indicates that systemic factors require swift and accurate resolution. Furthermore, the absence of a permanent service place and medical supplies hampers the availability of necessary routine services at the community level.

Support systems and the role of Puskesmas

Our findings showed that support from Puskesmas is essential to help enhance the performance of Posyandu, especially in urban areas. In examining the implications, it is crucial to consider the role of Puskesmas in the village healthcare system. The units have a well-defined operational role, serving as the frontline in addressing public health issues, disease prevention, and health promotion. However, this analysis highlights a significant deficiency in operating support for supervision, mentoring, logistics, and equipment for Posyandu. This lack affects the effectiveness and efficiency of the services provided, which stem from inadequate resource allocation and a lack of prioritization in assisting community PHC activities. As a result, and as cited in another study, the emphasis in both settings is frequently placed on curative health services rather than preventive measures [25].

The village head’s governance roles

The absence of active CHWs and the lack of a permanent service location for Posyandu provide clear information about the ineffectiveness of village governance. The study revealed that a lack of active CHWs is a significant factor for poor Posyandu performance, with unadjusted ORs indicating that Posyandus without active CHWs are 2.28 times more likely to perform poorly. This trend is consistent in rural and urban areas, with an even greater impact in rural areas (aOR = 2.79). Additionally, the absence of a permanent service location, crucial for effective service delivery, was also significantly linked to poor-performing Posyandus (aOR = 1.49), although this association was not statistically significant in rural areas (aOR = 1.39). These findings highlight the critical need for active participation from CHWs and the establishment of permanent service locations to improve the performance of health services at the village level.

This study highlights village heads’ strategic position in the organization of health programs at the village level. There are noticeable deficiencies in active CHWs and community participation in Posyandus, which points to weaknesses in program organization and support by village institutions. This issue could be attributed to village heads’ lack of understanding or capacity to manage health programs. Furthermore, in PHC, village heads’ roles are crucial to spearheading Posyandu operations, necessitating support in community mobilization, training, and mentorship from multisectoral teams [26,27].

The examination of village governance also underscores the importance of establishing village committees composed of community representatives [24,28,29]. These committees, which typically include community members, public health center staff, and volunteer organization representatives, play a crucial role in decision-making about village affairs, overseeing community health service delivery, and supporting volunteer workers. However, our data present a contrasting scenario in which village heads are perceived as detached and inconsequential to CHWs. This perception could contribute to the non-functionality of the intended role of governance. CHWs often find themselves limited to field-level issues, lacking the means to escalate these issues effectively.

To address these challenges, there is a pressing need for regulations that mandate the active participation of village leaders and committees in health initiatives. Such regulations should ensure that all residents understand their rights and responsibilities. Current regulations have only gone as far as transferring the ownership of Posyandu to village heads [30], allocating village funds for health [31], and failing to establish effective “village development committees.” Two primary concerns arise in this context. First, how could such regulations encourage village leaders to adopt a collaborative stance, inviting residents to participate in committees as part of village administration? This approach could balance the political interests of village leaders with the health improvement needs of the community. Second, strengthening civil society movements within these village volunteer assemblies is crucial, ensuring that they consistently advocate for their community’s interests.

Limitations

This study has several limitations. First, the use of an older dataset due to the unavailability of the most recent 2021 IFLS may not capture potential changes in population characteristics, healthcare practices, and epidemiological trends over time. However, given that the design of Posyandu has not evolved dramatically over the years, this study still provides a valuable perspective on the governance and systems issues at the Posyandu level. Further, the lessons learned from applying the health systems approach to understanding Posyandu as an integral part of the primary care system are instrumental. Second, recall bias may stem from the interview-led questionnaire employed for the IFLS data collection. To mitigate this, the IFLS survey limits the recall timeframe to two years before data collection. This, coupled with the fact that the interviewed CHWs were naturally embedded within Posyandu’s catchment area, their perception of the challenges was based on lived experience and may limit the recall bias.

Policy implications and recommendations

The significant implication of this study is that current policies, which view Posyandu (Integrated Health Service Posts) merely as a part of community-based efforts driven by the good intentions of community members to help each other, are overly simplistic. This perspective fails to recognize the complexity of health systems and underestimates the multifaceted roles and responsibilities required for effective health service delivery at the community level. This study suggests that relying solely on communal goodwill and voluntary participation is insufficient for the sustainable operation and success of Posyandu. This calls for a more comprehensive policy approach that acknowledges the need for structured support, adequate resources, and professional guidance to enhance the efficacy and impact of Posyandu services. This entails a shift from viewing Posyandu merely as a community initiative to recognizing it as an integral part of the national health system, requiring systematic support and integration with broader health policy objectives.

These findings underscore the heightened imperative for the Ministries of Home Affairs and Health to ensure robust oversight and involvement. As a practical implication, there is a necessity for advocacy on how the health systems approach should become the primary framework of thought in regional health planning and execution. Even though there are problems with bureaucracy and management coordinating Puskesmas [30] and there is no good governance [32], we need to look into options where village heads and higher levels of local government can manage cross-sectoral programs at the community level, such as hiring managers outside of sectoral bureaucracy [25,33]. This exploration is crucial for enhancing the efficiency and effectiveness of healthcare delivery at the grassroots level. Such an approach could circumvent the complexities of traditional bureaucratic systems and foster more dynamic and responsive healthcare management, aligning with the needs and realities of local communities.

This advocacy should integrate the health systems approach into the core strategies of regional health governance, which is in line with Indonesia’s health system transformation recently set by the Ministry of Health. Indonesia’s healthcare transformation is anchored by the Primary Service Transformation, which serves as its foundational pillar. This initiative primarily aims to bolster promotive and preventive activities. Additionally, it seeks to enhance health screening procedures and augment the capacity of primary care services, thereby ensuring comprehensive and accessible healthcare for all. Under the health transformation, CHWs are considered one of the main resources for community engagement and education, as well as in the delivery of Posyandu’s activities. Further, Indonesia’s reform efforts underscore the imperative of incorporating systemic perspectives to achieve comprehensive and sustainable healthcare outcomes. It involves promoting a comprehensive understanding of this approach among key stakeholders, including policymakers, health administrators, and practitioners at the regional level, echoing the Ministry of Health’s directives to create a more integrated and resilient healthcare system. Moreover, advocacy must address the need for adequate resource allocation, capacity building, and policy reform to support the health systems approach, which should be done at all levels and relevant sectors. The latter includes the district health offices, village-level government, offices, and the Ministry of Home Affairs that oversee sub-national level governance. These actors must facilitate cross-sectoral collaboration, enhancing the capabilities of health personnel, and ensuring that health initiatives are aligned with broader socioeconomic development goals, also in accordance with Indonesia’s strategic priorities for healthcare reform.

We recommend further research to focus on the positions of village heads and Puskesmas as governance structures within community activities. However, stakeholders convene to devise strategies to enhance the effectiveness of Posyandu activities for the community. This recommendation acknowledges the critical role of village heads and Puskesmas in shaping the governance landscape of community health initiatives. The emphasis on these roles underscores the need for a deeper understanding of how their involvement, or lack thereof, impacts the efficiency and success of Posyandu activities. Concurrently, the practical aspect of this recommendation highlights the immediacy of stakeholder engagement in formulating actionable strategies. This dual approach, theoretical exploration and practical application, is essential for developing a comprehensive understanding of the dynamics at play and for implementing effective interventions for community health improvement.

The findings from this study may also contribute to the understanding and strengthening of community-based health services in similar settings. For instance, success stories on the role of CHWs in improving healthcare access in other low-middle-income countries (LMICs) have cited limited support or supervision and quality control from local primary health centers [34,35]. Other studies on LMICs have also pointed out some gaps in the coordination and governance of integrated CHW-led programs [36-38]. However, these studies could benefit from using a similar health system lens, which may reshape the thinking on how to systematically assess the gaps in governance of similar community-led health services.

## Conclusions

Problems with operational funds, activity equipment, and the availability of routine places for service outlets are among the highest priorities in implementing Posyandu. Almost half of the Posyandus in this analysis were poor performers, with the numbers being more prevalent in rural areas. More interestingly, a lack of support from village heads received the lowest score. A lack of active community workers was the strongest determinant of poor-performing Posyandus. These shortcomings, coupled with the lack of governance roles of Puskesmas and village heads, elucidate the need for using health systems and governance frameworks in PHC. Stakeholders must convene and deliberate on a consensus regarding how both village heads and Puskesmas can collaboratively develop PHC systems, posting village heads to organize local-level resources in an effective governance system.

## Appendices

Supplementary file 1

Posyandu types and indicators based on the development stage

(1) The early stage (Pratama) is still in the early stages of establishment, has a small number of CHWs (less than five), and is not yet fully operational. (2) Intermediate Posyandu (Madya) carries out eight routine activities annually, with an average of five or more CHWs in charge. However, their coverage of the five primary public health activities is still low (less than 50%). (3) Advanced (Purnama) Posyandu carries out routine activities more than eight times per year, has five or more active CHWs, coverage of its five principal activities of more than 50%, can carry out development activities, and has received community-based health funding to support health activities at Posyandu. (4) Self-sufficient (Mandiri) Posyandus have all the features of an advanced Posyandu with the addition of receiving funding from business groups (businesses managed by the community).

The included question from the IFLS-5 questionnaire

The independent variables were derived from question A12: “Mention 3 (three) main problems faced by this health post in the last two years” of the “Book Pos” and the topic of integrated community health post (Posyandu) of the IFLS-5. The respondents who were CHWs responded to three of these questions: A = lack of funds; B = lack of medical supplies; C = lack of equipment; D = lack of active CHWs; E = lack of support from community health centers (Puskesmas); F = lack of support from villages or townships; G = no permanent place; H = lack of interest or participation; V = other; and W = no problem.

## References

[1] World Health Organization (2019). Report of the Global Conference on Primary Health Care: From Alma-Ata Towards Universal Health Coverage and the Sustainable Development Goals. https://apps.who.int/iris/bitstream/handle/10665/330291/WHO-UHC-IHS-2019.62-eng.pdf.

[2] Rasanathan K, Montesinos EV, Matheson D, Etienne C, Evans T (2011). Primary health care and the social determinants of health: essential and complementary approaches for reducing inequities in health. J Epidemiol Community Health.

[3] Sumriddetchkajorn K, Shimazaki K, Ono T, Kusaba T, Sato K, Kobayashi N (2019). Universal health coverage and primary care, Thailand. Bull World Health Organ.

[4] Assefa Y, Hill PS, Gilks CF, Admassu M, Tesfaye D, Van Damme W (2020). Primary health care contributions to universal health coverage, Ethiopia. Bull World Health Organ.

[5] Stenberg K, Hanssen O, Bertram M, Brindley C, Meshreky A, Barkley S, Tan-Torres Edejer T (2019). Guide posts for investment in primary health care and projected resource needs in 67 low-income and middle-income countries: a modelling study. Lancet Glob Health.

[6] Woldie M, Feyissa GT, Admasu B (2018). Community health volunteers could help improve access to and use of essential health services by communities in LMICs: an umbrella review. Health Policy Plan.

[7] Perry HB, Zulliger R, Rogers MM (2014). Community health workers in low-, middle-, and high-income countries: an overview of their history, recent evolution, and current effectiveness. Annu Rev Public Health.

[8] Kruk ME, Myers M, Varpilah ST, Dahn BT (2015). What is a resilient health system? Lessons from Ebola. Lancet.

[9] Wagenaar BH, Augusto O, Beste J (2018). The 2014-2015 Ebola virus disease outbreak and primary healthcare delivery in Liberia: time-series analyses for 2010-2016. PLoS Med.

[10] Omam LA, Metuge A (2023). Rapid response mechanism in conflict-affected settings of Cameroon: lessons learned from a multisector intervention for internally displaced persons. J Glob Health Rep.

[11] (2024). The World Bank In Indonesia. Overview. 6 Mar.

[12] (2024). Indonesia Ministry of Health. Transformasi Kesehatan Indonesia. Transformasi Kesehatan Indonesia.

[13] (2023). Posyandu is increasingly ready to serve people of all ages. https://ayosehat.kemkes.go.id/posyandu-semakin-siap-melayani-masyarakat-secara-menyuluh-.

[14] Leimena SL (1989). Posyandu: a community based vehicle to improve child survival and development. Asia Pac J Public Health.

[15] Saito A (2006). The development of the Posyandu: historical and institutional aspects. Critical and Radical Geographies of the Social, the Spatial and the Political.

[16] Rahayu SK, Toyamah N, Hutagalung SA, Rosfadhila M, Syukri M (2008). Qualitative Baseline Study for PNPM Generasi and PKH: The Availability and Use of the Maternal and Child Health Services and Basic Education Services in the Provinces of West Java and East Nusa Tenggara. SMERU Research Institute.

[17] Setiawan A, Christiani Y (2018). Integrated health post for child health (Posyandu) as a community-based program in Indonesia: an exploratory study. J Keperawatan Indonesia.

[18] Ministry of Health (2022). Guideline for Managing Posyandu (Pedoman Umum Pengelolaan Posyandu). Ministry of Health.

[19] Nazri C, Yamazaki C, Kameo S, Herawati DM, Sekarwana N, Raksanagara A, Koyama H (2016). Factors influencing mother's participation in Posyandu for improving nutritional status of children under-five in Aceh Utara district, Aceh province, Indonesia. BMC Public Health.

[20] Rahmawati ND, Sartika RA (2020). Cadres’ role in Posyandu revitalization as stunting early detection in Babakan Madang Sub-District, Bogor District. ASEAN J Community Engagement.

[21] Tumbelaka P, Limato R, Nasir S, Syafruddin D, Ormel H, Ahmed R (2018). Analysis of Indonesia’s community health volunteers (kader) as maternal health promoters in the community integrated health service (Posyandu) following health promotion training. Int J Community Med Public Health.

[22] World Health Organization (2007). Everybody’s Business: Strengthening Health Systems to Improve Health Outcomes: WHO's Framework for Action. https://books.google.com/books/about/Everybody_s_Business.html.

[23] Scott K, George AS, Ved RR (2019). Taking stock of 10 years of published research on the ASHA programme: examining India's national community health worker programme from a health systems perspective. Health Res Policy Syst.

[24] Karuga R, Kok M, Mbindyo P, Otiso L, Macharia J, Broerse JE, Dieleman M (2023). How context influences the functionality of community-level health governance structures: a case study of community health committees in Kenya. Int J Health Plann Manage.

[25] McCollum R, Limato R, Otiso L, Theobald S, Taegtmeyer M (2018). Health system governance following devolution: comparing experiences of decentralisation in Kenya and Indonesia. BMJ Glob Health.

[26] Rasanathan K, Atkins V, Mwansambo C, Soucat A, Bennett S (2018). Governing multisectoral action for health in low-income and middle-income countries: an agenda for the way forward. BMJ Glob Health.

[27] Patel G, Garimella S, Scott K, Mondal S, George A, Sheikh K (2017). Doing implementation research on health governance: a frontline researcher's reflexive account of field-level challenges and their management. Int J Equity Health.

[28] Ved R, Sheikh K, George AS, Vr R (2018). Village Health Sanitation and Nutrition Committees: reflections on strengthening community health governance at scale in India. BMJ Glob Health.

[29] Srivastava A, Gope R, Nair N (2016). Are village health sanitation and nutrition committees fulfilling their roles for decentralised health planning and action? A mixed methods study from rural eastern India. BMC Public Health.

[30] Ministry of Home Affairs (2011). Minister of Home Affairs Regulation Number 19 of 2011 on Guidelines for the Integration of Basic Social Services at Integrated Service Posts. https://peraturan.bpk.go.id/Details/111821/permendagri-no-19-tahun-2011.

[31] President of Indonesia (2023). Government Regulation No. 8 of 2016 on the Second Amendment to Government Regulation Number 60 of 2014 Concerning Village Funds Sourced From the State Revenue and Expenditure Budget. https://peraturan.bpk.go.id/Home/Details/5729/pp-no-8-tahun-2016.

[32] Madon S, Krishna S (2022). Theorizing community health governance for strengthening primary healthcare in LMICs. Health Policy Plan.

[33] Kesale AM, Kinyaga A, Mwakasangula E, Mollel H, Rashid SS (2023). Who makes health governance decisions at the local level? A cross sectional study from primary health facilities in Tanzania. Health Sci Rep.

[34] Zulu JM, Hurtig AK, Kinsman J, Michelo C (2015). Innovation in health service delivery: integrating community health assistants into the health system at district level in Zambia. BMC Health Serv Res.

[35] Tseng YH, Griffiths F, de Kadt J, Nxumalo N, Rwafa T, Malatji H, Goudge J (2019). Integrating community health workers into the formal health system to improve performance: a qualitative study on the role of on-site supervision in the South African programme. BMJ Open.

[36] Maru S, Nirola I, Thapa A (2018). An integrated community health worker intervention in rural Nepal: a type 2 hybrid effectiveness-implementation study protocol. Implement Sci.

[37] Shaw B, Amouzou A, Miller NP, Tafesse M, Bryce J, Surkan PJ (2016). Access to integrated community case management of childhood illnesses services in rural Ethiopia: a qualitative study of the perspectives and experiences of caregivers. Health Policy Plan.

[38] Seidenberg PD, Hamer DH, Iyer H (2012). Impact of integrated community case management on health-seeking behavior in rural Zambia. Am J Trop Med Hyg.

